# Substitution

**Published:** 1888-05

**Authors:** 


### SUBSTITUTION.

Dr. MITCHELL, in a letter to *Philadelphia Medical Times*, says: "In regard to substitution and adulteration, it must be admitted that in numerous cases the charge is a true one, and the evil is of growing dimensions. With the reduction in the margin of profits caused by the fierce business competition of the present day, comes the temptation to adulterate or substitute inferior quality. No condemnation can be too severe for the man who thus trifles with human life; and if he cannot carry on his business honestly, he had better abandon it and seek some other occupation.

"Again, the outcry is made that the physician is too apt to prescribe various remedies, more or less proprietary in character, put up by large manufacturing concerns and introduced by skilled advertising, and thus require the druggist to carry an endless variety of such articles in stock, many of which are seldom or only once called for, and thus remain a dead loss to the proprietor. But is the physician much to blame? True, he is sometimes imposed upon by the bland and suave canvasser, and the glowing printed endorsements of his professional brethren in favor of some new remedy—*vide* stenocarpine. But when he sees remedies in convenient and compact shape, of appearance much more elegant than those he can procure from the corner druggist, and of at least equal efficacy, is it to be wondered that he should prefer X., Y. or Z.'s manufactures to the oftentime imperfectly prepared remedies of the pharmacopœa?

" And why should the druggist complain? *As long as he keeps open store he must submit to the unalterable law of traffic, namely, the needs of the customers are to be supplied.* He will buy Lubin's extracts for Miss Jones, and Alfred Wright's for Miss Brown. Why should he not keep bromidia for Dr. A. and papine for Dr. B? Although he makes a great outcry about being obliged to carry so much stock, he in reality does it to a very limited extent, and outside of a few standard preparations, shifts the burden on his wholesale druggist and lets him carry the supply for him. Nearly all the large manufacturers have established depots for their goods in the principal cities, and the druggist very rarely lays in a stock outside of his actual present need, unless he is sure of a steady sale. And let him remember also that if he don't keep what is called for, some one else will, and his customers will be sure to go where their needs receive best attention.

"And here let a word be said for that much abused class, the modern manufacturers of pharmaceutical specialties. The medical and pharmaceutical profession owe to them a great debt. It is their industry and their capital which have developed the perfection of the coated-pill, and the compressed tablet, the pancreatic ferment and the scale pepsin, the smooth and palatable cod-liver oil emulsion, and the perfected extracts of malt. To their energy do we owe the modern methods of treating disease with the pre-digested and concentrated foods—a plan which has been the means of prolonging many valuable lives. They have spread the fame of American pharmacy over the entire globe, and established its supremacy against all competitors; therefore let them receive at least just recognition and honor for their labors."

